# Effect of handrubbing using locally-manufactured alcohol-based handrubs in paediatric wards in Harare, Zimbabwe

**DOI:** 10.1186/s13756-016-0166-8

**Published:** 2017-01-11

**Authors:** Muchaneta Gudza-Mugabe, Marcelyn T. Magwenzi, Hilda A. Mujuru, Mutsa Bwakura-Dangarembizi, Valerie Robertson, Alexander M. Aiken

**Affiliations:** 1 National Microbiology Reference Laboratory, Harare Central Hospital, Harare, Zimbabwe; 2 Department of Medical Microbiology, University of Zimbabwe-College of Health Sciences, Harare, Zimbabwe; 3 Department of Paediatrics and Child Health, University of Zimbabwe-College of Health Sciences, Harare, Zimbabwe; 4 Parirenyatwa Hospital, Harare, Zimbabwe; 5 London School of Hygiene and Tropical Medicine, London, UK; 6 Biomedical Research and Training Institute, Harare, Zimbabwe

**Keywords:** Hand hygiene, Contamination, Gram negative bacteria, Nosocomial infection, Paediatric, Alcohol-based hand rub

## Abstract

We assessed bacterial contamination of hands of adults present in paediatric wards in two tertiary-care hospitals in Harare, Zimbabwe and the microbiologic efficacy of locally-manufactured alcohol-based hand rub (ABHR). During unannounced visits, samples were collected using hand-print and hand-rinse methods. Samples were collected from 152 individuals (16 nurses, 10 doctors, 28 students, 86 parents/guardians, 12 others). Contamination of hands with Gram-negative bacteria was found in 91% of adults tested with a mean of 14.6 CFU (hand-rinse method; IQR 3–65), representing a high risk for transmission of pathogens potentially leading to nosocomial infections. A single application of ABHR under controlled conditions achieved an average of 82% (or 0.72 log) reduction in detectable counts. Amongst 49 *Enterobacteriaceae* isolates from hands, 53% were resistant to gentamicin and 63% were resistant to cefpodoxime. Use of ABHR represents an attractive intervention for reducing nosocomial infections in this setting.

## Background

Health-care workers’ (HCWs) hands have frequently been found to be colonized with potential pathogens during routine patient care [1]. These organisms survive if hand hygiene by HCWs is inadequate or omitted entirely. Infections are transmitted [2] when contaminated hands come in direct contact with another patient or an object used for patient care [3, 4].

Zimbabwe has been undergoing strenuous economic challenges for more than a decade. Amongst other difficulties, this has led to intermittent water supply in many hospitals. Alcohol-based hand-rub (ABHR) is recommended as an effective method of hand decontamination during routine patient care [5, 6] and is locally-produced in many hospitals in low-income countries [6].

This study was a cross-sectional evaluation ahead of a planned trial of an ABHR-based intervention. We focused on the detection of Gram-negative *Enterobacteriaceae* on the hands of adults to determine whether this potentially represented a major vector for transmission of antibiotic-resistant *Enterobacteriaceae* between paediatric patients in this setting.

## Methods

Study participants were recruited from the paediatrics wards of two tertiary government-sector hospitals in Harare, Zimbabwe. These hospitals have established hospital infection programs, but no routine data on hand-hygiene performance was being collected at the time of the study.

After relevant ethics approvals, all adults present in wards on unannounced study dates were approached for participation. After informed consent was obtained, one hand (either left or right) was tested before and after ABHR use [7]. Four fingers of the selected hand were lightly pressed to an agar plate to make a print (hand-print method). The same hand was then rinsed in a bag containing 200 ml of sterile water by inserting the entire hand, shaking vigorously and rubbing fingers and thumb together for 15 s (hand-rinse method). Plastic bags (Zipwave, Multix, Australia) were sterilized between uses by immersion in boiling water. From each hand-rinse sample, 100 ml of liquid was filtered using paper with 0.45 μm apertures (Millipore, UK). A 0.5 MacFarland suspension of ATCC 25923 *E.coli* and sterile water were used as controls.

Alcohol-based hand rub prepared at Parirenyatwa Hospital Pharmacy following the WHO guidelines [8] was used. For quality control in production, the percentage alcohol content was routinely tested using a hydrometer [6]. Additionally, a sample of ABHR from the same production batch as used in this study was analysed by spectrography in University Hospital Geneva Pharmacy, Switzerland in June 2015. Approximately 5 ml of ABHR was dispensed onto both the hands of the participant and the investigator showed how to rub the hands together according to standard WHO method for hand-cleansing [9]. After ABHR use, samples were immediately collected from the opposite hand using the same methods as described above.

Samples from both methods were cultured aerobically on MacConkey agar at 37 °C for 18–24 h. Plates were read and the Gram-negative colonies were counted manually. Fifty oxidase-negative isolates identified to the level of family/genus and species using API10S strips (Biomerieux, France). *Enterobacteriaceae* isolates were tested for resistance to six antibiotics according to the British Society of Antimicrobial Chemotherapy standard method [10]. Analysis of data was performed using a Microsoft Excel spreadsheet and statistical tests were performed using STATA v14.1.

## Results

The study took place over a 2-week period in May 2015. Samples were collected from 152 individuals in the participating wards (16 nurses, 10 doctors, 86 parents/guardians, 28 students, 12 others). Before the use of ABHR, Gram-negative bacteria were detected on the hands of 91% (138/152) of adults tested by either the hand-print or hand-rinse method. The mean recovery rate for Gram-negative bacteria was high, with a geometric mean of 6.8 CFU (IQR 1–21) and 14.6 CFU (IQR 3–65) detected by the hand-print and hand-rinse method respectively. Immediately after ABHR use, the mean recovery rate for Gram-negative bacteria from the opposite hand was 1.2 CFU (IQR 0–2) and 2.4 CFU (IQR 0–5 CFU) by hand-print and hand-rinse methods (Table 1).

**Table 1:** Gram-negative CFU counts before and after use of ABHR

		Geometric mean Gram-negative CFU (IQR)			
	*n*	BEFORE use of ABHR		AFTER use of ABHR	
Population tested		Hand-print	Hand-rinse	Hand-print	Hand-rinse
All participants	152	6.8 (1–21)	14.6 (3–65)	1.2 (0–2)	2.4 (0–5)
Dominant hand “Before”	116	6.8 (0–22)	13.7 (2–65)	1.4 (0–3)	2.4 (0–5)
Non-dominant hand “Before”	13	24.1 (12–65)	30.2 (8–160)	1.6 (0–6)	3.5 (0–27)
Unknown dominant hand	23	3.1 (1–6)	13.4 (2–51)	0.5 (0–1)	1.8 (0–3)
Nurse	16	5.9 (2–11)	13.0 (5–193)	1.2 (0–1)	1.8 (0–7)
Doctor	10	1.0 (0–2)	4.8 (0–14)	0.1 (0–0)	2.3 (0–9)
Student	28	5.1 (0–13)	10.2 (2–24)	0.4 (0–1)	1.4 (0–2)
Parent/guardian	86	9.4 (1–40)	18.5 (3–153)	1.9 (0–4)	3.0 (0–6)
Other/unknown professional group	12	5.6 (1–11)	7.2 (2–22)	0.8 (0–2)	0.8 (0–2)
Male	27	3.5 (0–8)	10.5 (1–53)	0.6 (0–1)	1.9 (0–6)
Female	119	7.3 (1–24)	15.6 (3–82)	1.4 (0–3)	2.4 (0–5)

There was some indication that on the hand tested before the use of ABHR, the recovery rate for Gram-negative bacteria was higher on the non-dominant hand (mean for hand-rinse method = 33.4 CFU; *n* = 14) than on the dominant hand (mean = 13.7 CFU; *n* = 116) but the difference was not statistically significant (Wilcoxon rank sum test; *p* = 0.21).

Amongst sub-groups of people, the recovery of Gram-negative bacteria before use of ABHR was lowest amongst doctors (mean for hand-rinse method = 4.8 CFU; *n* = 10) and highest amongst parents/guardians (mean = 18.5 CFU; *n* = 86), although this difference did not reach statistical significance (Wilcoxon rank sum test; *p* = 0.15).

The reductions in Gram-negative bacteria counts with use of ABHR were 82% (or 0.75 log) according to the hand-print method and 84% (or 0.78 log) according to the hand-rinse method. Spectrographic analysis of ABHR used showed the correct alcohol concentration (83%; acceptable range 75-85%), but hydrogen peroxide was not detected (expected concentration 0.125%).

Amongst Gram-negative oxidase-negative bacterial isolates recovered from participants hands in this study, identification using API10S indicated that 49/50 were *Enterobacteriaceae,* with the most likely genus being Klebsiella (*n* = 21), Citrobacter (*n* = 15), Pantoea (*n* = 8), Escherichia (*n* = 4) and Yersinia (*n* = 1). Susceptibility testing indicated that antibiotic resistance was widespread in these isolates, including resistance to gentamicin (26/49; 53%) and cefpodoxime (34/49; 63%) (Table 2).

**Table 2:** Susceptibility testing amongst Enterobacteriaceae isolated (*n* = 49)

Antibiotic (disc used)	Susceptible (%)	Intermediate (%)	Resistant (%)
Ampicillin (10 μg)	1 (2%)	-	48 (98%)
Gentamicin (10 μg)	22 (45%)	1 (2%)	26 (53%)
Ciprofloxacin (1 μg)	14 (29%)	11 (22%)	24 (49%)
Cefpodoxime (10 μg)	12 (25%)	3 (6%)	34 (69%)
Chloramphenicol (30 μg)	29 (59%)	-	20 (41%)
Ertapenem (10 μg)	43 (88%)	2 (4%)	4 (8%)

## Discussion

In paediatrics wards in government hospitals in Harare, we found that contamination of the hands of HCWs and parents with Gram-negative bacteria was extremely common (91% with detectable bacteria) and with a high burden of organisms, representing a high risk for transmission of pathogens which might cause infection. Interestingly, bacterial loads were found to be lowest amongst doctors and highest amongst the parents/guardians of paediatric patients and on non-dominant hands, though these were based on small numbers. We did not investigate whether specific ward-based tasks increased levels of contamination. Community-based work with mothers in Tanzania found that various activities including cleaning children’s faeces increased faecal indicator bacteria levels on hands [11]. The organisms we isolated were frequently resistant to antibiotics routinely used for treatment, representing a direct risk to patients [12]. The frequent resistance to cefpodoxime, an agent used to screen for the presence of Extended-Spectrum β-lactamases, and gentamicin suggests that transmission of this form of resistance might be occurring via the hands of adults in these wards [13]. A single application of locally-manufactured ABHR achieved substantial but not complete reduction in detectable Gram-negative bacteria counts. Ideally, we should have tested equal numbers of dominant and non-dominant hands prior to ABHR use—this may have led to underestimation of the efficacy of ABHR. We cannot say with certainty whether the reductions in Gram-negative bacteria on hands demonstrated in this study would reflect on clinical outcomes in routine practice.

Repeated use of ABHR could be used as a strategy to prevent cross-infection between patients via the hands of staff and visitors in this setting [5]. System changes to enable this practice must be addressed in Zimbabwean hospitals where ABHR use has not yet become a standard of care. In addition to making ABHR more widely available, there is an important need to instruct parents and HCWs in hospitals in Zimbabwe on the essential role of hand hygiene in care of hospitalized children.

## Abbreviations

ABHR: Alcohol-based hand rub
CFU: Colony-forming units
HCWs: Health-care workers
IQR: Inter-quartile range
WHO: World Health Organisation
